# Biodiesel Production From Lignocellulosic Biomass Using Oleaginous Microbes: Prospects for Integrated Biofuel Production

**DOI:** 10.3389/fmicb.2021.658284

**Published:** 2021-08-12

**Authors:** Anjani Devi Chintagunta, Gaetano Zuccaro, Mahesh Kumar, S. P. Jeevan Kumar, Vijay Kumar Garlapati, Pablo D. Postemsky, N. S. Sampath Kumar, Anuj K. Chandel, Jesus Simal-Gandara

**Affiliations:** ^1^Department of Biotechnology, Vignan’s Foundation for Science, Technology and Research, Guntur, India; ^2^Department of Chemical, Materials and Production Engineering, Università degli Studi di Napoli Federico II, Naples, Italy; ^3^LBE, INRAE, Université de Montpellier, Narbonne, France; ^4^College of Agriculture, Central Agricultural University, Imphal, India; ^5^ICAR-Indian Institute of Seed Science, Mau, India; ^6^ICAR-Directorate of Floricultural Research, Pune, India; ^7^Department of Biotechnology and Bioinformatics, Jaypee University of Information Technology, Waknaghat, India; ^8^Laboratory of Biotechnology of Edible and Medicinal Mushrooms, Centro de Recursos Naturales Renovables de la Zona Semiárida (CERZOS-UNS/CONICET), Buenos Aires, Argentina; ^9^Department of Biotechnology, Engineering School of Lorena (EEL), University of São Paulo (USP), Lorena, Brazil; ^10^Nutrition and Bromatology Group, Department of Analytical and Food Chemistry, Faculty of Food Science and Technology, University of Vigo, Ourense, Spain

**Keywords:** biodiesel, bioethanol, greenhouse gas, lignocellulosic materials, liquid fuels

## Abstract

Biodiesel is an eco-friendly, renewable, and potential liquid biofuel mitigating greenhouse gas emissions. Biodiesel has been produced initially from vegetable oils, non-edible oils, and waste oils. However, these feedstocks have several disadvantages such as requirement of land and labor and remain expensive. Similarly, in reference to waste oils, the feedstock content is succinct in supply and unable to meet the demand. Recent studies demonstrated utilization of lignocellulosic substrates for biodiesel production using oleaginous microorganisms. These microbes accumulate higher lipid content under stress conditions, whose lipid composition is similar to vegetable oils. In this paper, feedstocks used for biodiesel production such as vegetable oils, non-edible oils, oleaginous microalgae, fungi, yeast, and bacteria have been illustrated. Thereafter, steps enumerated in biodiesel production from lignocellulosic substrates through pretreatment, saccharification and oleaginous microbe-mediated fermentation, lipid extraction, transesterification, and purification of biodiesel are discussed. Besides, the importance of metabolic engineering in ensuring biofuels and biorefinery and a brief note on integration of liquid biofuels have been included that have significant importance in terms of circular economy aspects.

## Introduction

Biodiesel is one of the prospective renewable fuels produced from different plant oils, animal fats, waste oils, and microbial lipids (Banerjee et al., 2019a; Kumar et al., 2020c). The exorbitant cost of vegetable and non-edible oils, requirement of acreage, and lower yield of oil have forfeited the purpose of using these feedstocks for biodiesel production (Nathan et al., 2019). In general, these feedstocks incur 75% of the overall production cost of biodiesel production (Subramanian et al., 2010). The development of cost-effective feedstocks coupled with judicious utilization of waste substrates could be a viable option to reduce the cost of the process. In addition, encouraging circular economy process is gaining significant interest to reduce the wastage of resources (Kumar and Banerjee, 2019).

Oleaginous microorganisms (OMs) are capable of utilizing inexpensive feedstocks [agro-residues, lignocellulosic substrates (LCSs)] and waste substrates for higher lipid accumulation (Kumar et al., 2020d). Besides, employing OMs could realize the potential of circular economy and development of cost-effective processes. For instance, crude glycerol, a by-product of the biodiesel industry, can be supplemented as a carbon source for lipid accumulation (Kumar et al., 2020e). Thereafter, these lipids can be converted to fatty acid methyl esters (FAMEs) and glycerol that again can be recycled. Envisaging this process could realize the viability of the process and hence cost-effective biodiesel production using agro-residues, glycerol, and other waste substrates (Chandel et al., 2020).

Production of OMs is easy to scale up, does not require land and acreage, and results in higher lipid accumulation within shorter incubation times with desirable lipid composition. In case of oleaginous microalgae, they can assimilate atmospheric carbon dioxide into lipid synthesis that ultimately helps in carbon sequestration (Kumar et al., 2017a). Tapping the potential of OMs and lignocellulosic biomass (LCB), this paper is mainly aimed to disseminate the usage of LCS for biodiesel production using OMs. Besides, an integration biofuel production such as bioethanol and biodiesel can also be produced through circular economy process.

## Conventional Feedstocks Used for Biodiesel Production

Feedstocks’ price has been identified as the most significant factor affecting the overall economic feasibility of biodiesel market (Zhang et al., 2003; Dorado et al., 2006). Approximately, feedstock cost incurs 70–75% of the total biodiesel production cost (Haas et al., 2006). Thus, to provide viable biodiesel, the raw material cost must be considered a key parameter (Dorado, 2008). For an efficient biodiesel production process, usage of low-cost feedstock, low production costs, and large production scale should be considered as an important requisite. The most common feedstocks used for biodiesel production are vegetable oils derived from edible plants, but the ethical awareness about the use of food as fuel and their high cost have encouraged research efforts to search for low-cost non-food feedstocks.

Criteria for effective utilization of non-food feedstocks should supply raw material for an entire year, value-added products for biochemicals, favorable fatty acid composition, low agricultural inputs (water, fertilizer, pesticides), high oil content, and adaptability to local growing conditions (rainfall, soil type, latitude, etc.) (Patel et al., 2017). Feedstock-derived biodiesel meeting at least the majority of the above criteria represents the most effective alternative to petrodiesel. Since last several years, various feedstocks have been explored for biodiesel production, which can be categorized into four groups: vegetable oil (edible and non-edible), waste or recycled oil, animal fat, and OMs (Kumar et al., 2019).

### Vegetable Oil (Edible and Non-edible)

Vegetable oil as biodiesel feedstock has a number of advantages such as transportability, easy availability, sustainability, high combustion ability, minor sulfur content, lesser aromatic content, and eco-friendly nature (Demirbas et al., 2016), while the major drawbacks include highly viscous nature and unsaturated hydrocarbon chain reactivity (Demirbas, 2009a, b). Vegetable oil includes edible and non-edible oil. Edible vegetable oil is the first and main feedstock for the production of biodiesel. Edible oil-based biodiesel shares in excess of 95% world biodiesel production (Sajjadi et al., 2016). The wide utilization of edible oils as biodiesel feedstock is due to their availability, easy processing, and biodiesel quality obtained from them.

The most widely used edible vegetable oil feedstocks for global biodiesel production are rapeseed oil used in Europe and Canada, sunflower oil used in Europe, palm oil in Southeast Asia, and soybean oil in the United States (Demirbas et al., 2016). The other edible oils that were explored for biodiesel production are corn, cottonseed, coconut, peanut, linseed, sesame, almond, etc. However, it is improbable for these to be used for commercial biodiesel production because of their high production and trading price. Global biodiesel production has mostly derived from rapeseed oil and sunflower oil, which contribute 84 and 13% of the total production, respectively (Atabani et al., 2012).

Palm oil contributes 1%, while others including soybeans account for 2% of the global biodiesel production (Atabani et al., 2012). However, edible oils cannot be served as a long-standing choice for potential feedstock because of issues associated with it, out of which significant increase of their price and food vs. fuel crisis are major constraints. There are also environmental concerns associated with the production of edible oil-based biodiesel to fulfill the market demand. There is a need to enhance feedstock production that leads to deforestation, consumption of ample agricultural lands, damage of vital soil resources, among others (Sajjadi et al., 2016). Non-edible vegetable oil has been found as a potential alternative to edible oil for biodiesel production. They have certain advantages over edible vegetable oil feedstock, as they can grow on wasteland, they have a low cost of growing, and there is no food vs. fuel issue. In the last few years, biodiesel production from various non-edible oil sources has been extensively studied (Jena et al., 2010; Sharma and Singh, 2010; Wang et al., 2011).

Many non-edible oil-producing plants were investigated for biodiesel feedstock, which includes *Jatropha curcas* (jatropha), *Pongamia pinnata* (karanja), *Hevea brasiliensis* (rubber), *Madhuca indica* (mahua), *Ricinus communis* (castor), *Azadirachta indica* (neem), *Nicotiana tabacum* (tobacco), *Gossypium hirsutum* (cotton seed), *Simmondsia chinensis* (jojoba), *Moringa oleifera* (moringa), *Mesua ferrea* (Nahor), *Simarouba glauca* (Simarouba), *Sapindus mukorossi* (soap nut), and *Schleichera oleosa* (kusum) (Singh et al., 2020). Non-edible vegetable oil is a better alternative but has some constraints in the path to be suitable for biodiesel feedstock such as long growing period, requirement of ample land for sufficient production, and variation in lipid quality upon changing the plant species, climate, season, and geography (Kumar et al., 2020a).

### Waste or Recycled Oil

Any oils that are unsuitable for its original purpose after being used owing to the presence of impurities or loss of original properties are termed waste oil. It could be a good feedstock alternative for biodiesel production, as it is much cheaper compared to vegetable oil. In the global scenario, there is generation of a huge quantity of waste oils from household and industrial sectors. A study reported approximately 7,00,000 to 10,00,000 tons/year generation of waste cooking oil in the EU (Supple et al., 2002). In Canada, yellow grease [waste cooking oil with free fatty acid (FFA) content less than 15%] generation was reported about 1,20,000 tons/year (Zhang et al., 2003).

Disposal of large amounts of waste oil is a serious environmental problem in many countries. Utilization of waste oil for biodiesel production has potential to solve this problem. However, the presence of many impurities such as FFAs, polymer, and many other compounds in the waste oils makes their conversion to biodiesel a complex process. Moreover, logistics and collection infrastructure are not well developed and hence could be a hurdle due to scattered sources (Sajjadi et al., 2016). Extensive literature has been published on the utilization of waste oil as biodiesel feedstock (Yaakob et al., 2013; Hajjaria et al., 2017; Fonseca et al., 2019; Talavari et al., 2020).

### Animal Fat

Similar to waste oil, animal fat is a cheaper source for biodiesel production and has an environmental advantage as using the waste. In the United States, animal fats account for one-third of the produced fats and oils (Sajjadi et al., 2016). The production cost of biodiesel from refined animal fats was estimated to be 0.4–0.5 USD per liter, which is lower compared to the cost of vegetable oil transesterification (around 0.6–0.8 USD per liter) (Balat, 2011). Generally, major animal fats include tallow, lard, poultry fat, fish processing industry-generated fat, and fat from the leather industries.

Industrial-scale biodiesel production using some of the animal fat feedstocks has already been reported (Schörken and Kempers, 2009). The animal fat-derived biodiesel has higher caloric value, cetane number, and oxidation stability (Ramírez-Verduzco et al., 2012; Adewale et al., 2015), but due to the high amount of FFAs and saturated fatty acids, the transesterification process is more challenging (Sajjadi et al., 2016). Limited availability of animal fat compared to the world’s biodiesel demands is further added to its disadvantages as a biodiesel feedstock.

### Oleaginous Microbes

Oleaginous microorganisms are the microbes that accumulate lipid that is more than 20% of their dry weight based on stress condition, i.e., higher carbon and low nitrogen sources (Ratledge and Wynn, 2002; Meng et al., 2009), and may reach up to 70% or more providing the stress conditions (Rossi et al., 2011). These microbes use various renewable materials and convert them into microbial oil, which is utilized further to produce biodiesel through transesterification (Ma et al., 2008; Pinzi et al., 2013). The microbial oil is also termed as single-cell oil. The first single-cell oil was commercially produced way back in 1985 using filamentous fungus *Mucor circinelloides*, but its utilization for biodiesel production was not in thought at that time (Ratledge, 2004).

The general scheme of biodiesel production from OMs is culturing of microbes, biomass harvesting, drying, lipid extraction, and transesterification of the obtained lipid (Kumar et al., 2013). OM-based biodiesel production has a number of advantages such as overcoming the food vs. fuel crisis, shorter incubation time compared to plant and animal resources, independence of lipid production from variation of season, climate, and geography (Gujjala et al., 2017), and year-round culturing of OMs and microbial oil production. Additionally, the microbial oil has comparable composition and caloric value to those extracted from animal and plant resources, and low viscosity. Furthermore, OMs can be utilized to convert inexpensive agro-industrial wastes and even municipal wastes to microbial oil with similar quality as many high-value lipids (Cho and Park, 2018). Inexpensive lignocellulosic wastes may serve as a potential source for microbial oil production. However, lower lipid yield and tolerance against the degradation products generated out of lignocellulosic’s pretreatment process are among the major bottlenecks for economical production of microbial oil or single-cell oil (Jin et al., 2015). OMs belong to different microbial families, namely, microalgae, yeast, filamentous fungi or molds, and bacteria (Ma, 2006).

Currently, microalgae-based biodiesel is the focus of research, as it may provide sufficient oil as feedstock for global consumption and produce biodiesel yields far higher than those recorded per hectare from plant feedstock (Zuccaro et al., 2020). Furthermore, they have the potential to mitigate land use and food vs. fuel conflicts and be cultivated in habitats that are not favorable for energy crops. They are also able to reduce the greenhouse effect *via* CO_2_ sequestration (Chisti, 2007). The lipid content in microalgae usually varies from 20 to 50% of the dry cellular weight, and it may increase up to 90% providing certain conditions (Adachi et al., 2011; Papanikolaou and Aggelis, 2011; Table 1). Considering the usual value of 30% dry weight for microalgal cell lipid content resulted in microalgal oil production of 4.5–7.5 t/ha/year (Tsukahara and Sawayama, 2005). It is much higher in comparison to the soybean, rapeseed, palm, and jatropha oil production, which are 0.4, 1.4, 1.6, 3.6, and 4.1 t/ha/year, respectively (Chisti, 2007; Lam and Lee, 2012).

**TABLE 1:** Lipid production by oleaginous microalgae using lignocellulosic substrates.

Oleaginous microalgae	Substrate	Lipid production	References
*Chlorella pyrenoidosa*	Rice straw enzymatic hydrolysate	1.55 g/L^a^ 53.6%^b^	Li et al., 2001
*Chlorella protothecoides*	Cassava starch enzymatic hydrolysate, corn powder enzymatic hydrolysate	2.14 g/L^a^ 22 ± 55.2%^b^	Xu et al., 2006; Wei et al., 2009; Lu et al., 2010
*Chlorella vulgaris*	Wheat bran enzymatic hydrolysate	N.A g/L^a^ 0.6 ± 0.9%^b^	El-Sheekh et al., 2012
*Schizochytrium limacinum*	Sweet sorghum juice (squeezed by a mill)	2.15 ± 4.95 g/L^a^ 55.3 ± 70.5%^b^	Liang et al., 2010
*Schizochytrium mangrovei*	Food waste hydrolysate	3.52 g/L^a^ 16.4%^b^	Pleissner et al., 2013
*Chlorella protothecoides*	Sugarcane bagasse hydrolysate	5.8 g/L^a^ 34.0%^b^	Mu et al., 2015
*Nannochloropsis* sp.	Palm oil mill effluent	3.2 g/L^a^ 11.0%^b^	Cheirsilp et al., 2017
*Auxenochlorella protothecoides*	Organosolv/steam explosion pretreated Birch biomass	5.7 g/L^a^ 66 %^b^	Patel et al., 2018
*Auxenochlorella protothecoides*	Organosolv/steam explosion pretreated Spruce biomass	5.3 g/L^a^ 63 %^b^	Patel et al., 2018
*Schizochytrium* sp.	Sugarcane bagasse hydrolysate	N.A g/L^a^ 45.15%^b^	Nguyena et al., 2018

*^a^Lipid yield, ^b^Lipid content (% w/w).*

Based on the modes of nutrition, microalgae are classified into heterotrophic, autotrophic, and mixotrophic, which imply that they have different metabolic pathways. In heterotrophic mode, organic compounds would serve as carbon sources that can be utilized for growth, which is advantageous in terms of higher productivity and concentration. In autotrophic regime, microalgae obtain carbon source from carbon dioxide, which is reduced with the help of light energy and releases O_2_. Predominantly, most of the microalgae confine to this category with minimal requirement of vitamins and organic compounds for growth. Mixotrophy mode comprises utilizing both organic carbon source and CO_2_ as substrates for growth by photosynthesis and cell respiration pathways. Cell growth pattern in mixotrophy is similar to heterotrophic mode as the latter growth is the sum of autotrophic and heterotrophic modes and results in higher productivity (Aratboni et al., 2019).

High production cost of microalgae-based biodiesel is the main hurdle for microalgae to be used as a suitable feedstock. As per literature, production cost of microalgal oil is 5.3–8.0 USD per liter (Pinzi et al., 2013). The major factors for the high production cost of microalgal oil include the biomass productivity, lipid content, production scales, and cost of oil recoveries from biomass (Balat, 2011). Heterotrophic growth and mixotrophic growth have great advantage of higher biomass and lipid productivity, but providing inexpensive suitable organic carbon source is one of the major limitations and hence one of the major factors for high production cost of microalgae-based biodiesel. Lignocellulosic wastes that are available in plenty presented as one of the options for cheap organic carbon source for microalgal growth (Chandel et al., 2020).

Molds or filamentous fungi are OMs that can store lipid up to 80% of their biomass composition (Pinzi et al., 2013). Many species of oleaginous fungus have been studied in recent years. These microbes can be grown on renewable carbon sources including lignocellulosics-based and usually their lipid has more amounts of unsaturated fatty acids compared to yeast (Hui et al., 2010; Papanikolaou and Aggelis, 2011; Economou et al., 2012; Papanikolaou and Aggelis, 2019). High lipid content and utilizing renewable carbon source for their growth favored oleaginous fungus to be a potential candidate of biodiesel feedstock (Table 2). Additionally, in contrast to microalgae, they can grow in the traditional bioreactors that will result in the reduction in production (biomass and oil) cost. Regardless, fungal oil utilization to produce biodiesel is still at laboratory scale.

**TABLE 2:** Lipid production by oleaginous molds using lignocellulosic substrates.

Oleaginous molds	Substrate	Lipid production	References
*Mortierella isabellina*	Corn stover enzymatic hydrolysate, sweet sorghum	0.016 ± 0.11 mg/gds^a^ 29.47 ± 38.36%^b^	Economou et al., 2011; Ruan et al., 2012
*Microsphaeropsis* sp.	Wheat straw	80 mg/gds^a^ 10.2%^b^	Peng and Chen, 2008
*Aspergillus oryzae*	Wheat straw + bran	62.9 mg/gds^a^ N.A.%^b^	Hui et al., 2010
*Colletotrichum* sp.	Rice straw + wheat bran	68.2 mg/gds^a^ N.A.%^b^	Dey et al., 2011
*Alternaria* sp.	Rice straw + wheat bran	60.3 mg/gds^a^ N.A.%^b^	Dey et al., 2011
*Mucor Circinelloides* Q531	Mulberry branches	42.43 ± 4.01 mg/gds^a^ 28.8 ± 2.85%^b^	Qiao et al., 2018
*Phanerochaete Chrysosporium ATCC 24725*	Wheat bran, corn straw and glucose mixture	>40%^b^	Liu et al., 2019
*Mucor circinelloides*	Hydrolyzed whey permeates	32%^b^	Chan et al., 2020

*^a^Lipid yield (mg/g dry substrate), ^b^Lipid content (% w/w).*

Many yeast species (approximately 30 species) have already been recognized as oleaginous, and this list is continuously increasing (Luis et al., 2016). Mostly, these species have suitability for large-scale fermentation and are also amenable to genetic improvement (Athenaki et al., 2018). Oleaginous yeasts are found to store lipid up to 70% under conditions with nutrient limitation (Subramanian et al., 2010). They have faster growth compared to microalgae and contain triglycerides as the majority of the lipids (Leesing and Karraphan, 2011). Yeast oil production is found to be costlier than vegetable oil production because of the expensive commercial substrate; hence, selecting oleaginous yeast species that can grow using cheap substrate is crucial (Table 3).

**TABLE 3:** Lipid production by oleaginous yeast using lignocellulose substrates.

Oleaginous yeast	Substrate	Lipid production	References
*Cryptococcus curvatus*	Corncob hydrolysate, sweet sorghum bagasse enzymatic hydrolysate	10.83 g/L^a^ 61 ± 73.26%^b^	Liang et al., 2012; Chang et al., 2015
*Lipomyces starkeyi*	Corncob acid hydrolysate, sugarcane bagasse acid hydrolysate	8.1 g/L^a^ 26.9 ± 55%^b^	Huang et al., 2014; Xavier et al., 2017
*Rhodosporidium toruloides*	Jerusalem artichoke, cassava starch enzymatic hydrolysate	14 ± 39.6 g/L^a^ 43.3 ± 63.4%^b^	Zhao et al., 2010; Wang et al., 2012
*Rhodotorula glutinis*	Corncob acid hydrolysate; wheat straw acid hydrolysate	1.4 ± 5.5 g/L^a^ 11.86 ± 36.4%^b^	Liu et al., 2015
*Trichosporon cutaneum*	Corncob acid and enzymatic hydrolysates, Diluted acid pretreated and biodetoxified corn stover	9.8 ± 12.3 g/L^a^ 32.1 ± 40%^b^	Liu et al., 2012; Gao et al., 2014
*Yarrowia lipolytica*	Sugarcane bagasse and rice bran hydrolysate	5.2 ± 6.68 g/L^a^ 48 ± 58.5%^b^	Tsigie et al., 2012
*Cryptococcus vishniaccii MTCC232*	Paper mill sludge	7.8 g/L^a^ 53.4%^b^	Deeba et al., 2016
*Cutaneotrichosporon cutaneum*	Corn stover hydrolysate	4–5 g/L^a^	Wang et al., 2016
*Vishniacozyma psychrotolerans*	Groundnut shell hydrolysate	46%^b^	Deeba et al., 2017
*Rhodotorula glutinis*	Cassava bagasse hydrolysate	10.42 g/L^a^ 51%^b^	Liu et al., 2018
*Candida phangngensis PT1-17*	Switchgrass hydrolysate	9.8 g/L^a^	Quarterman et al., 2018
*Rhodotorula paludigenum*	Corncob hydrolysate	3.29 g/L^a^ 58%^b^	Chaiyaso et al., 2019
*Meyerozyma guilliermondii*	Sugarcane bagasse hydrolysate	37.99 ± 0.003%^b^	Ananthi et al., 2019
*Pichia kudriavzevii*	Rice husk hydrolysate	28.57 ± 0.009%^b^	Ananthi et al., 2019
*Naganishia albida*	Biowaste hydrolyzed by microbes	13.5 g/L^a^ 20%^b^	Sathiyamoorthi et al., 2019
*Cutaneotrichosporon dermatis*	Corn stover hydrolysate	20.36 g/L^a^ 56%^b^	Yu et al., 2020

*^a^Lipid yield, ^b^Lipid content (% w/w).*

Lignocellulosic waste is one of the cheap carbon sources that have been used for producing yeast oil using various yeast species in a number of studies. Recently, potentiality of oleaginous yeast for lipid production has been reviewed by Sreeharsha and Mohan (2020). However, yeast oil availability for commercial utilization is not yet reached.

Bacteria are having the properties of high growth rate and ease of genetic manipulation, which can be positively exploited for high microbial oil production. Generally, bacteria are not known for high lipid accumulation except some species, for example, *Rhodococcus*, *Streptomyces*, and *Mycobacterium* (Zuccaro et al., 2020). The actinomycetes are reported to have accumulation of fatty acid up to 70% of their cell dry weight (Kalscheuer et al., 2006). Genetic engineering in bacteria resulted in many high-lipid yielding species. It was reported that genetic changes in *Escherichia coli* led to 2.5 g/L of fatty acid production (Lu et al., 2008). Furthermore, many bacteria can be grown using lignocellulosic-based substrate as a carbon and energy source.

The bacteria *Rhodococcus opacus*, which has the ability to store more than 80% triglycerides (triglyceride transesterification leads to biodiesel or FAME formation) of its dry biomass, was grown solely on cob waste and achieve triglyceride production up to 59.26 mg/L/day (Alvarez and Steinbüchel, 2002). Also, *R. opacus* DSM1069 can degrade and live on lignin-derived compounds like coniferyl alcohol (Eggeling and Sahm, 1980). Utilization of lignocellulosic hydrolysates by numbers of other bacterial species including genetically engineered ones has also been reported in the literature for lipid production (Kumar et al., 2020b; Table 4). There are also reports on microbial oil production using algal–bacterial consortia (Zhang et al., 2020). Nonetheless, bacterial oil production is in infant stages at laboratory scale only, and commercial biodiesel production from bacteria is still a far distant story.

**TABLE 4:** Lipid production by oleaginous bacteria using lignocellulose substrates.

Oleaginous microbes	Substrate	Lipid production	References
*Gordonia* sp. DG	Orange waste	71%^a^	Gouda et al., 2008
*Rhodococcus opacus* PD630	Orange waste	83%^a^	Voss and Steinbuchel, 2001
*R. opacus* Xsp8	Kraft hardwood (hydrolysate)	45.8%^a^	Kurosawa and Sinskey, 2013
*Rhodococcus opacus*	Oxygen-pretreated Kraft lignin	14.2 %^a^	Wei et al., 2015
*Rhodococcus opacus*	Effluent from lignocellulosic pretreatment	26.9^a^	Goswami et al., 2017

*Lipid content (% w/w).*

## Lignocellulosics and Their Potential for Biodiesel Production

Lignocellulosics are considered among the most promising alternatives to fossil resources (Keegan et al., 2013; Shaheena et al., 2019), as they are generated from available atmospheric CO_2_, water, and sunlight through biological photosynthesis. They are sustainable sources of organic carbon and account for excess of 90% of worldwide plant biomass, corresponding to annual biomass of approximately 200 × 10^9^ tons (Kuhad and Singh, 1993). LCB resources and their division are not well-defined. They can be categorized into four classes, viz., agricultural residues, forest residues, herbaceous grass and weeds, and industrial and municipal waste (Kumar et al., 2020c). Agricultural residues are observed to be the largest lignocellulosic resource (Yousuf, 2012). They include all the food crop residues and non-food crop residues. Wheat straw, rice straw, corn residues, and sugarcane bagasse are the major contributors of the agricultural residues.

Worldwide annual availability of wheat straw, rice straw, corn straw, and sugarcane bagasse residues are estimated to be 354.34, 731.3 205, 128.02, and 180.73 million tons (Mt), respectively (Saini et al., 2015). In Indian scenario, annual generation of the total agricultural crop residues is approximately 500 Mt, while around 140 Mt are surplus crop residues, out of which 90 Mt are surplus cereal residues (Bhuvaneshwari et al., 2019). Many countries especially European countries and the United States cultivate non-food crops or energy crops for biofuel production; the residues generated out of these crops are parts of agricultural residues. A study suggested that approximately 2.5 M ha of land were used for energy crops’ cultivation with 80–85% of rapeseed crops by European Union member countries in the year 2005 (Yousuf, 2012).

Herbaceous grasses and weeds can become agriculture residues, as they come out from agricultural fields and/or marginal lands. The second largest lignocellulosic resource is considered to be forest residues (Yousuf, 2012; Kumar et al., 2021). Usually, forest residues are referred to the parts of trees that are inappropriate for saw logs such as branches, dead wood, foliage, and treetops (Hossain and Badr, 2007). Tree fallings, residues from wood processing, and recycled wood can also be included in this group. Food industries and many other industries based on plants and plant products produce lignocellulosic wastes that can be utilized for microbial oil production. Municipal wastes also contain lignocellulosic components and hence can serve as a feedstock for microbial oil production.

Considering the commercial perspective of the biodiesel, generally, 40–80% of total production cost is incurred on substrate acquisition. Lignocellulosics, being the most abundant biorenewable biomass resource in the world (Somerville et al., 2010; Zhou et al., 2011) and economically cheap, can serve as a potential alternative for the cost-effective microbial oil production (Yousuf, 2012) leading to biodiesel production. From an economic point of view, lignocellulosics can be produced quickly and at a lower cost than other biofuel feedstocks (Huber, 2008). On the contrary, biofuels, particularly biodiesel, polymers, and biorefinery production from LCB are a great challenge owing to biomass recalcitrance of the lignin polymer (Himmel et al., 2007; Zhou et al., 2011). To reduce the recalcitrance, there is a requirement of pretreatment process that may include chemical, physical, or biological method. Prior to the pretreatment of LCB, the biomass needs to be processed by subjecting it to milling, which might cost up to 5% of the investment. Pretreatment of lignocellulosics resulted in the generation of various compounds as by-products such as furan aldehyde (furfural, 5-hydroxymethylfurfural), aliphatic carboxylic acids (acetic acid, formic acid, levulinic acid), and aromatic carboxylic acids (cinnamic and benzoic acid derivatives), which are detrimental to the growth of the microorganism involved in the bioprocessing for lipid production (Huang et al., 2009; Jönsson and Martín, 2016; Poontawee et al., 2017; Llamas et al., 2020; Valdés et al., 2020).

Effects of these inhibitors on OMs’ physiology have been studied, and interestingly, it was observed that OMs are tolerant to inhibitors, and some are even having degradation properties such as *Trichosporon cutaneum*, which converts furfural and hydroxymethylfurfural (HMF) to their corresponding alcohol. In addition, it degraded formic acid and acetic acid (Chen et al., 2009; Wang et al., 2016; Poontawee et al., 2017; Valdés et al., 2020). Furthermore, acetic acid was observed to be the substrate and stimulator for lipid production by a number of OMs (Poontawee et al., 2017; Llamas et al., 2020). It was also found that microbes having a natural tolerant property against the inhibitors are not very efficient and rather have a restricted ability to convert sugars into lipid-rich biomass (Valdés et al., 2020). This can be overcome by genetically manipulating the OMs, which is not much difficult due to evolution and advancement of genetic engineering technology.

To address this problem, extensive research efforts have been made; as a result, several promising biorefinery technologies and demonstration plants using non-food cellulosic biomass feedstocks have been increasing (Cherubini and Strømman, 2011; Chandel et al., 2018).

### Structural Features of Lignocellulosics for Biodiesel Production

Lignocellulosic biomass are composed of cellulose, hemicellulose, and lignin polymers as major components with percent dry weight of 30–35%, 20–25%, and 15–20%, respectively (Haghighi et al., 2013; Uthandi et al., 2021). This composition of lignocellulosics varies on the variation of their geographical location, growing conditions, and age (Pérez et al., 2002). Kumar et al. (2020e) have presented an exhaustive list of LCBs with their composition as dry weight percentage of lignin, cellulose, and hemicellulose. Biodiesel production from LCBs consists of four important steps such as delignification, saccharification, fermentation with OMs for higher lipid synthesis, and subsequent conversion to transesterification (Zhao et al., 2008; Kumar et al., 2017c).

Lignin polymer is a non-carbohydrate fraction that consists of phenyl propionic moieties and situated in the primary cell wall (Yousuf, 2012; Banerjee et al., 2019a). Lignin is made up of coniferyl alcohol, coumaryl alcohol, and sinapyl alcohol units bonded with varied ether bonds and imparts tensile strength, resistance against microbial attack, and impermeability. Owing to its (lignin) close association with cellulose microfibrils, the former acts as a barrier to microbial and enzymatic hydrolysis (Chintagunta et al., 2017). Lignin content varies in different feedstocks that need to abate through a delignification step for easy access of cellulose and hemicellulose polymers (Galbe and Zacchi, 2002).

In plant cell structure, cellulose polymer occupies a major proportion, which is made up of D-glucose moieties linked by β-1,4-glycosidic bonds with a degree of polymerization of 10,000 or even higher. Cellulose polymers are packed into microfibrils through Van der Waals forces and hydrogen bonds. Owing to this bonding and structural confirmity, cellulose manifests tensile strength to cell wall, chemical stability, crystallinity, and resistance to microbial degradation (Himmel et al., 2007; Banerjee et al., 2017). Due to the cellulose inherent property, LCBs show recalcitrance behavior (Banerjee et al., 2019b).

Unlike cellulose polymer, hemicellulose is made up of several monomer units such as D-galactose, D-mannose, D-xylose, D-glucose, D-glucuronic acid, L-arabinose, and 4-*O*-methyl-D-glucuronic that have an amorphous structure with a degree of polymerization lower than 200 (Chandel et al., 2020). Hemicellulose is an abundant polymer next to cellulose, which varies in composition from one feedstock to another. For example, in softwood, hemicelluloses are composed of gluco-mannan, while in grasses and agro-residues (straw), hemicelluloses are made up of xylan (Kumar et al., 2020a). Hemicelluloses form a complex network by linking with lignin and cellulose microfibrils that impart mechanical strength and sensitivity to thermal and chemical treatments (Banerjee et al., 2019a).

Efficient hydrolysis (saccharification) requires removal of at least 50% of hemicellulose for increased cellulose digestibility. However, treatment parameters should optimize meticulously for abatement of furfurals and HMFs that inhibit the fermentation process. With an objective of maximum sugar recovery, in general, the pretreatment conditions are compromised, and depending on the treatment, hemicellulose may be obtained as either solid or liquid fractions (Banerjee et al., 2017).

## Bioconversion of Lignocellulosic Biomass to Biodiesel Production

The intricacy in the use of the LCB is due to the presence of physical barriers formed by the lignin that prevents the penetration of hydrolytic enzymes and access the cellulosic fraction of the biomass (Kucharska et al., 2018). Thus, the processing of LCB to biodiesel is complicated and includes several unit operations: pretreatment/depolymerization, hydrolysis/saccharification, culturing the OMs for lipid production, and transesterification. Each unit process is discussed extensively with a special emphasis on the various techniques involved in it.

### Lignin Depolymerization

Pretreatment of the LCB is done to remove lignin and achieve maximum hydrolysis of cellulose and hemicellulose components for the production of fermentable sugars. This process reduces the crystallinity of the cellulose, increases the porosity, and improves the access of chemicals and hydrolytic enzymes toward the holocelluloses for effective hydrolysis of biomass (Jacob et al., 2016). A multitude of pretreatment methods have been developed in the last few decades where an ideal pretreatment process should have the features such as complete saccharification of the holocelluloses, minimum loss of sugars, by-product formation, and energy consumption. There are various pretreatment techniques that include physical, chemical, physicochemical, and biological pretreatments for lignin removal from LCB (Table 5).

**TABLE 5:** Various methods of pretreatment and their effect of the lignocellulosic biomass.

Pre-treatment	Technique	Effect on biomass	Yield	References
Mechanical ∙ Chipping ∙ Milling ∙ Grinding	Vibratory ball milling, Ball milling, Hammer milling, Colloid milling, Wet disk milling	Improves digestibility of the biomass and increases surface area Reduction in crystallinity of cellulose and degree of polymerization	Increase of 5–25% in yield of sugars after hydrolysis	Barakat et al., 2015
Microwave assisted size reduction	High-pressure microwave pretreatment is operated in closed reactors within the temperature range from 150 to 250°C	Molecular collisions due to dielectric polarization generates thermal energy that leads to disruption of the lignocellulosic structure	Highest methane yield of 221 mL⋅g–sub^–1^ was obtained from microwave pretreated *Hyacinthus* spp.	Zhao et al., 2017
Steam explosion	Exposure of lignocellulosics to hot steam up to 160–260°C, 0.69–4.38 MPa pressure for few minutes	Solubilization of hemicellulose leads to formation of acid that catalyze hydrolysis of soluble fractions Improved exposure of cellulose to hydrolytic chemicals and enzymes	Facilitates upto 90% of enzymatic hydrolysis	Grous et al., 1986
Hot water/aquasolv/uncatalyzed solvolysis/aqueous fractionation/hydrothermolysis	Exposure of lignocellulosics to 200–230°C and high pressure for few minutes	40–60% of the biomass gets dissolved	4–22% of cellulose, 35–60% of lignin and entire hemicelluloses is digested No formation of toxic inhibitory compounds if pH is maintained between 4 and 7	Kumar et al., 2009
Ammonia fiber explosion (AFEX)	Biomass (1 kg) is treated with hot liquid ammonia (1–2 kg) under high pressure at 90°C for 30 min, followed by sudden release of the pressure	Alters the structure of the biomass Increases the water-holding capacity of the biomass and its digestibility	Increases the yield upon hydrolysis by six folds	Alizadeh et al., 2005
Acid	Sulfuric acid (0.2–2.5 wt%) is mixed with biomass and the pretreatment is carried out either at 180°C or above for 5 min or 120°C for 30–90 min	Hydrolyzes hemicellulose to its constituent monomers Removal of hemicellulose and lignin promotes efficient hydrolysis of cellulose	Acid pretreatment in combination with efficient hydrolysis, 100% recovery of sugar from biomass is achievable	Mosier et al., 2005
Alkaline	Lignocellulosic biomass is soaked in the solution containing hydroxides of calcium/sodium, potassium/ammonia	Promotes degradation of glycosidic side chains and ester linkages, leading to structural alteration of lignin, partial decrystallization and swelling of cellulose. Solvation of hemicelluloses and saponification of intermolecular ester linkages, leads to enhanced porosity of the biomass.	At optimal conditions (1.5% NaOH solution, 144 h incubation time and 20°C), 60% lignin removal and 80% release of hemicellulose sugar (as xylose) took place from wheat straw.	Chen et al., 2012
CO_2_ explosion	At conditions above critical point of 31°C and 7.39 MPa, CO_2_ behaves as a supercritical fluid and has access to pores in biomass CO_2_ reacts with moisture to form carbonic acid, which further catalyzes biomass degradation	Explosive release of pressure leads to the disruption of hemicellulose and cellulose structure and increases the overall accessible area to enzymes.	Supercritical CO_2_ pretreatment results in 75% yield of glucose relative to its theoretical yield.	Maurya et al., 2015
Ozonolysis	The terminal oxygen of ozone is electron deficit and selectively attacks substrates rich in electrons such as lignin, while carbohydrates mostly remain unaffected	By ozonolysis, degradation is mostly limited to lignin, while hemicellulose is slightly affected and cellulose is unaffected	Removal of lignin (60%) through ozonolysis, led to fivefold increase enzyme hydrolysis rate of wheat straw.	Li et al., 2015
Organosolv	It operates at 90–120°C (for grasses) and 155–220°C (for wood), 25–100 min incubation time Solvent and catalyst concentration varies with the type of feedstock.	The internal lignin and hemicellulose bonds will be broken simultaneously	90% hydrolysis of softwood pulp cellulose took place in 48 h using the solvent, ethanol.	Haghighi et al., 2013
Biological	Deconstruction of lignin structure in the cell wall using microbes and/or enzymes under ambient conditions	Microorganisms and the enzymes are known for lignin and hemicellulose removal with a very little effect on cellulose	Pretreatment of Bermuda grass by *Cyathus stercoreus* resulted in 63–77% of delignification which leads to improved production of fermentable sugars	Chaturvedi and Verma, 2013
Ionic liquid	Conditions maintained for carrying out IL pretreatment are : 100–150°C and biomass to ionic liquid ratio of 1:10 (w/w)	This method simultaneously dissolve lignin and carbohydrates After pretreatment, cellulose can be separated by addition of anti-solvents followed by centrifugation and filtration Significant changes occurs in cellulose crystallinity, structure, composition, and surface characteristics of the biomass	Enhancement of biomass digestibility More than 90% conversion of cellulose to sugars takes place	Singh et al., 2009
Oxidative	Addition of oxidizing agents like O_2_/H_2_O_2_/peracetic acid to the biomass suspended in water	The technique involves the removal of hemicelluloses and lignin to improve the accessibility of hydrolytic agents towards cellulose	Peracetic acid hydrolysis of poplar and sugarcane bagasse increased reducing sugar yield upto 98%	Tan et al., 2010

The mechanical/physical pretreatment methods, viz., irradiation, pyrolysis, extrusion, and pulsed electric field, reduce the crystallinity, particle size, and degree of polymerization and increase the available surface area for effective action of hydrolytic enzymes. Being energy intensive, the physical pretreatment is not viable at industrial scale. Chemical pretreatment methods include alkaline (NaOH, KOH, K_2_CO_3_, CaO, and MgO), acid (H_2_SO_4_, H_3_PO_4_, and HCl, zeolite, and SiO_2_-Al_2_O_3_ oxides), organosolv (methanol, acetone, and ethylene glycol), and ionic liquid (IL) {1-butyl-3-methylimidazolium chloride [(Bmim)Cl]} pretreatment (Baruah et al., 2018).

Physicochemical pretreatment is an integration of physical and chemical methods, and various methods included under this pretreatment are ammonia fiber explosion (AFEX), steam explosion, CO_2_ explosion, and hydrothermal methods. Furthermore, the biological pretreatment of biomass is conducted using either microorganisms or enzymes. It requires low energy and mild operation conditions. A decrease in lignin content enhances the yield of fermentable sugars from holocelluloses. Through pretreatment, a significant fraction of lignin will be removed from the biomass that upon recovery is generally utilized as fuel for generation of heat and power. But there are certain fungal species and oleaginous bacteria that can utilize lignin as substrate for supporting their growth and lipid production (Kosa and Ragauskas, 2012).

Besides using suitable pretreatment methods to reduce the lignin content, research has been carried out to study the effect of lignin biosynthesis genes on the amount and composition of lignin. Various parameters such as modifications in the target gene and the degree of downregulation of the enzyme activity, efficiency of the silencing construct utilized, size of the gene family, and the redundancy within the gene family influence the degree of lignin reduction, for instance, modifying the genes encoding for transcription factors, oxidative enzymes, etc., and reducing the activity of steps involved in lignin biosynthetic pathway, starting from phenylalanine ammonia-lyase (PAL) up to cinnamyl alcohol dehydrogenase (CAD) results in variation in the lignin content and composition (Wang J. P. et al., 2018). Moreover, downregulation of the steps from cinnamate 4-hydroxylase (C4H) up to cinnamoyl-CoA reductase (CCR) drastically reduces the lignin content when compared with downregulation of ferulate 5-hydroxylase (F5H), caffeic acid *O*-methyltransferase (COMT), and CAD. Downregulation of the caffeic acid *O*-methyltransferase gene modestly decreased the lignin content, reduced the syringyl:guaiacyl lignin monomer ratio, improved forage quality, and increased the ethanol yield by 38% in switchgrass (Fu et al., 2011). Reduced lignin content along with variation in the H/G/S ratios in the LCB affects the biomass processing efficiency. For example, increased level of H units reduces the length of lignin polymer and enhances the lignin removal from the biomass (Sykes et al., 2015). Besides, the processing efficiency of the biomass can also be enhanced by incorporating certain molecules, for example, ferulic acid in CCR-deficient trees results in the formation of acetal bonds in the lignin polymer, which are easily cleaved in acidic biomass pretreatment (Van Acker et al., 2014).

### Saccharification of Lignocellulosic Biomass

Saccharification is usually preceded by pretreatment of lignocellulosics to convert carbohydrate polymers efficiently into fermentable sugars. The efficiency of the process predominantly depends upon the pretreatment method, as improper maintenance of operating conditions during the process may lead toward the production of compounds having an inhibitory effect on the downstream processes. Commonly used techniques in hydrolysis of the cellulose fraction of biomass are chemical and enzymatic methods.

The chemical hydrolysis of polysaccharides in the biomass can take place in the presence of acid under high temperatures. It does not require pretreatment of the biomass and takes place at high rates. The drawbacks related to this method are formation of furfurals, low yield of sugars, and less economical feasibility of the process. The process can be made economical only through development of effective acid recovery techniques. Besides, the enzymatic hydrolysis is performed at low temperatures (45–50°C) and pressure and mild pH; high yield of fermentable sugar is obtained with low by-product formation without causing corrosion of equipment (Kucharska et al., 2020). Thus, high concentrations of enzymes are essential for cellulose hydrolysis, and their production needs knowledge of efficient microbial strains and economically feasible techniques.

Cellulose and hemicellulose conversion is catalyzed by cellulase and hemicellulase, which are complex enzymes. The subunits of cellulose include endoglucanases, exoglucanases, and β-glycosylases. Endoglucanase is involved in random digestion of internal β-1,4-glycosidic bonds, exoglucanase cleaves cellulose from either reducing or non-reducing ends, and β-glucosidase hydrolyzes cellobiose into glucose. Similarly, xylan-degrading enzymes include endoxylanases, β-xylosidases, α-glucuronidase, α-arabinofuranosidase, and acetoxylan esterase. Among the endo-β-1,4-xylanase hydrolyses, the β-(1,4) linkages of the xylan give rise to xylooligosaccharides, which will be further hydrolyzed into xylose units by β-xylosidase (Kumar et al., 2017c). Synergistic action of cellulases and hemicelluases will promote the conversion rate of holocelluloses into simple sugars. As each enzyme present in the cocktail has different optimal conditions, it is very difficult to determine optimal conditions for enzyme cocktail (Lopes et al., 2018).

### Lipid Biosynthesis

The hydrolysate obtained through the saccharification process is used as carbon source by various microbes such as *Gordonia* sp., *Mycobacterium tuberculosis*, *Chlorella* sp., *Rhodosporidium toruloides*, *Mortierella isabellina*, etc. (Kumar et al., 2020c). Most of these OMs lack the cellulase and xylanase activity, and hence, they cannot directly utilize the polysaccharides present in the biomass. Thus, there is every need to hydrolyze the polysaccharides into fermentable sugars such as glucose and xylose and supplement them to the microbe as carbon source. OMs have the capacity to convert glucose and similar sugars to pyruvate, which is further used in the synthesis of lipids. The three physiological phases of *de novo* biosynthesis include (i) growth phase under balanced conditions, (ii) oleaginous phase under nitrogen-limited conditions, and (iii) reserved lipid turnover phase that occurs after depletion of carbon compounds in the growth medium. The carbon source is converted in cell mass through glycolysis and pentose phosphate pathway, but the depletion of essential nutrients induces oil accumulation. Furthermore, during the lipid turnover phase, the triacylglycerol (TAG) is degraded for the generation of energy required for the maintenance of the cell. Approximately 0.32 g of lipid/g of glucose and 0.34 g of lipid/g of xylose can be produced by these microbes (Ratledge, 1988). Furthermore, strains such as *Rhodosporidium* and *Pseudozyma* were reported to consume xylose, glucose, and fructose and xylose, glucose, and arabinose respectively (Patel et al., 2015; Tanimura et al., 2016). Besides, Wang Q. et al. (2018) and Spagnuolo et al. (2019) observed enhanced accumulation of saturated fatty acids upon employing consortia of OMs.

Apart from the concentration of nutrients, other factors responsible for lipid accumulation are temperature, pH, culture agitation, and dissolved oxygen. It was reported that some microalgae accumulate high amounts of TAG under nitrogen-deficient conditions (Yodsuwan et al., 2017). Several works reported that the presence of ammonium nitrogen in the medium favors lipid synthesis, whereas a few reported the improved lipogenesis in the presence of organic nitrogen (Fakas et al., 2008). Moreover, limitations of minerals, viz., iron, magnesium, zinc, phosphorus, nitrogen, in the culture medium induce lipid biosynthesis. It was also reported that >30°C, neutral and basic pH, and 200–300 rpm enhance lipid production (Papanikolaou and Aggelis, 2011; Chaiyaso et al., 2019). Furthermore, reactive oxygen species (ROS) produced under stress may result in lipid peroxidation and denaturation of protein and DNA, but under balanced level, it promotes lipid production (Zhang et al., 2020).

**FIGURE 1:**
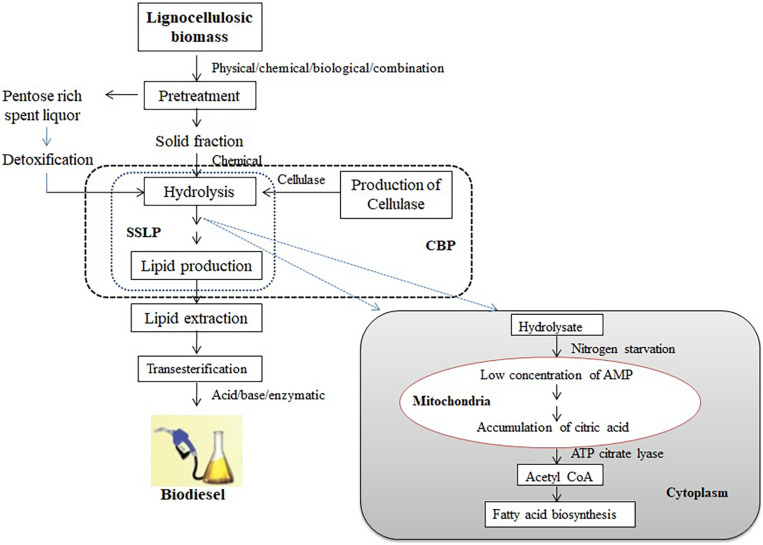
Schematic diagram of lipid biosynthesis from lignocellulosic biomass using oleaginous microorganisms.

Oleaginous microorganisms have the capacity of producing acetyl-CoA and nicotinamide adenine dinucleotide phosphate (NADPH) that are necessary for the synthesis of fatty acids. Excess carbon and nitrogen-deficit conditions induce the upregulation of AMP deaminase (AMPD), which cleaves adenosine monophosphate (AMP) to inosine monophosphate (IMP) and ammonia. Eventually, the concentration of AMP and activity of NAD^+^ (NADP^+^)-depended isocitrate dehydrogenase (ICDH) will be downregulated. In mitochondria, in the presence of aconitase, the accumulated isocitrate will be isomerized to citrate, which will be transported into the cytoplasm in exchange for malate. The OMs exclusively synthesize ATP citrate lyase, which will convert citrate into acetyl-CoA and oxaloacetate. The acetyl-CoA is used as a precursor for the synthesis of fatty acid, whereas oxaloacetate will be converted into malate in the presence of malate dehydrogenase and transported into mitochondria (Ratledge and Wynn, 2002; Figure 1).

Lipid biosynthesis by OMs from lignocellulosics can be carried out through separate hydrolysis and lipid production (SHLP) where hydrolysis and lipid production take place separately in two steps; simultaneous saccharification and lipid production (SSLP) where saccharification and lipid production take place simultaneously in a reactor; and consolidated bioprocessing (CBP) where cellulase production, hydrolysis, and lipid production take place in one step (Figure 1). SHLP is characterized by inhibition of cellulase by glucose, leading toward a reduction in the yield of simple sugars. In the case of SSLP, the sugars released due to hydrolysis get assimilated by microorganisms, thereby reducing the inhibitory effect of glucose. SSLP is more economical than SHLP, as the process is completed in a single vessel, but processing needs a compromise with temperature, as the temperature requirements are different for hydrolysis and lipid production, which is the disadvantage of the process (Olofsson et al., 2008).

An industrially viable strain that can produce cellulase with high titer for hydrolyzing cellulose and even accumulate lipid is either isolated or designed by genetic engineering for its application in CBP. The strain is designed by incorporating genes responsible for cellulose degradation and high lipid accumulation. These OMs can be grown on the biomass hydrolysate for production of lipids by either solid state or submerged fermentation processes. The lipid yield produced by various OMs grown on lignocellulosic hydrolysate has been illustrated in Tables 4, 5. There are various strategies to improve the lipid yield from the OMs grown on lignocellulosic hydrolysate (Santek et al., 2018). They include:

• Development of strain that has industrial viability: Through metabolic engineering (ME), the strain should be developed in such a way that it should produce a high yield of lipids, be tolerant toward inhibitors generated during delignification of lignocellulosics, and exhibit heterologous expression of cellulase.
• Construction of efficient bioreactor design: The reactor should be configured at very low capital and operational costs.
• Improved pretreatment for lignocellulosics: Cost-effective pretreatment process with limited inhibitor formation and enhanced hydrolysis should be employed.
• Effective utilization of by-products formed during the process: During the process of biodiesel formation, there is a scope of generation of several by-products such as lignin, sophorolipids, pigments. These by-products can generate an additional income in the lipid biorefinery.
• Advances in the downstream process: Development of an efficient method for large-scale extraction of lipids in a cost-effective way. Extraction of lipid from cell wet biomass is economical than extraction from dry biomass, as the cost incurred upon drying of biomass can be saved.

Apart from using various strategies for enhancing lipid production, advanced techniques such as microfluidics can be used to optimize the process parameters for production of lipids, transesterification, and quality assessment of blended biodiesel. Researchers have observed the synthesis of energy-rich lipid under yellow light (580 nm) (Shih et al., 2015). As the growth was not congenial, researchers exposed *Cyclotella cryptica* to alternative blue and yellow light for 15 and 8 h, respectively, which led to rapid proliferation and production of higher neutral lipids. Yeh et al. (2016) developed a microfluidic platform with a large surface-to-volume ratio that drives the chemical reaction by enhancing the material interface. Soybean oil and methanol are the components that participate in the transesterification process. This methanol is passed coaxially into the pool of the oil wherein the methanol is surrounded by the oil, resulting in the reduction in ratio of methanol:oil (3:1). At 1, 1:2, and 1:3 ratios of methanol:oil, the oil conversion was 100, 99.5, and 98.6%, respectively. The results infer that the oil:methanol is an essential parameter for enhancing the transesterification.

On the other hand, Kosa et al. (2018) used Duetz microtiter plate system in combination with Fourier transform infrared spectroscopy and multivariate analysis for high-throughput screening of efficiency of more than 100 Mucoromycota strains for production of high- and low-value lipids.

Upon considering the role of proteomics/transcriptomics for enhancing the production of lipids, in *Nannochloropsis gaditana*, the macronutrient stress is associated with lipid remodeling. Significant modifications occurred in some lipid-related proteins, including increased expression of diacylyglycerol acyltransferase-2 (DGAT) and lipid body proteins under N-starved conditions. Nitrogen starvation reduces growth and protein and chlorophyll contents concurrently increase neutral lipids, carbohydrates, and secondary carotenoids in various species. The effects of P deprivation are linked to remodeling of the lipid profile, where phosphorus-containing lipid classes are substituted for non-phosphorus lipids (Mühlroth et al., 2017).

Upon considering various OMs, viz., yeasts, fungi, microalgae, and bacteria, yeasts were regarded as the best candidate for higher lipid accumulation owing to their fast growth, high lipid accumulation, and easy scale-up (Younes et al., 2020). The lipid accumulated by the yeast generally contains fatty acids such as palmitic acid, myristic acid, oleic acid, stearic acid, linolenic acid, and linoleic acid (Fabiszewska et al., 2019). In order to obtain the lipid content from the microbes, there is a need that one should adopt an appropriate extraction method.

## Lipid Extraction and Transesterification

In order to extract the lipid from the microbe, the primary requisite is the disruption of the cell wall. Various techniques such as high-pressure homogenization, bead mill, ultrasound, pulsed electric field, osmotic shock, subcritical water hydrolysis, microwaves, enzymatic hydrolysis, autolysis, and chemical hydrolysis are generally used for cell disruption (Khot et al., 2020). Besides, hydroxyl radicals, nanoscalpels, and photocatalytically active amino clay-conjugated TiO_2_ are being used for cell disruption (Dong et al., 2016; Chintagunta et al., 2020). Furthermore, solvent extraction, pressing and solvent-integrated extraction, supercritical extraction, elevated temperature and pressure solvent extraction, electroporation-assisted extraction, ultrasound-assisted extraction, and ionic liquid co-solvent extraction are used for extracting the lipids from the OMs. In the entire process of biodiesel synthesis, lipid extraction alone will incur up to 90% of the cost; hence, in order to make the process economical, green solvent and green extraction techniques that can save time and energy and reduce solvent consumption are being used (Kumar et al., 2017b).

Even though the oleaginous biomass is rich in lipids, it also contains a large amount of proteins and carbohydrates. For instance, soybeans comprise 20%(wt.) lipid, 20%(wt.) carbohydrates, and nearly 40%(wt.) proteins (Maddi et al., 2018). Even the lipid-rich strains of microalgae contain significant amounts of non-lipid component. In spite of applying the biomass remnants obtained after lipid extraction to the soil, it can be processed for the generation of value-added products such as gasoline and methane. The dry algal biomass residue can be pyrolyzed to oils that are suitable as transportation fuels. The wet biomass can be processed through hydrothermal means and anaerobic digestion for the production of diesel, gasoline, and methane. As neither the algal biomass nor the remnants contain lignin, the obtained bio-oil will not contain any phenolic oligomers. But the proteins in the algal biomass/remnants will be converted to pyrroles, indoles, and poly-heteroaromatic compounds that need to be removed to prevent poisoning of the catalysts or nitrogen oxide emissions during combustion. In contrast, the pyrolysis of algal biomass in the presence of zeolite causes the biomass to denitrify and deoxygenate and produce aromatic hydrocarbons. The aromatics are high-value chemicals that are responsible for enhancing the octane rating of gasoline. This process opens a new avenue for converting algal biomass directly into fuels and high-value chemicals.

After extraction, the lipids must be converted into simple alkyl esters (biodiesel) through a process known as transesterification during which the viscosity of the lipids will be reduced. During the transesterification process, the lipid reacts with alcohol such as ethanol and methanol in the presence of a catalyst to form esters and glycerol (Kumar et al., 2019). Depending upon the catalysis mechanism, transesterification is classified into chemical-catalyzed reaction, non-catalyzed reaction, or enzyme-catalyzed reaction.

Chemical-catalyzed reactions are further classified into heterogeneous and homogeneous reactions. The heterogeneous reaction takes place in the presence of acid or alkali catalyst in solid state, and examples for these catalysts are calcium-based metal oxides and sulfated zirconia (Aransiola et al., 2014). Similarly, the homogeneous reaction takes place in the presence of acid or alkali catalyst in liquid state, and examples for such catalysts are hydrochloric acid, sulfuric acid, sodium hydroxide, and potassium hydroxide (Ong et al., 2014). Though the alkali-based transesterification process takes place quickly, it is not viable in case of feedstocks with higher FFAs due to the formation of soap. On the other hand, disadvantages with acid-based transesterification are longer incubation time, corrosive nature, higher energy consumption, glycerol purification costs, etc.

Non-catalyzed transesterification reaction occurs quickly under supercritical conditions, and the product obtained through the process can be separated easily without generation of waste. The demerits of the process are the requirement of high temperature (250–400°C) and pressure (10–30 MPa), which is very expensive (Stamenković et al., 2011). To address the problems related to the chemical-catalyzed and non-catalyzed reactions, the researchers explored enzymatic transesterification that operates under mild conditions and has high substrate specificity (Christopher et al., 2014). Shimada et al. (2002) reported 98.5% of FAME conversion by enzymatic transesterification (immobilized lipase from *Candida antarctica*) from docosahexanoic acid.

### Physicochemical Properties of Lignocellulosic-Based Oleaginous Fatty Acid Methyl Esters

Fatty acid methyl esters suitability is mainly determined through the produced FAME’s physicochemical properties, which have to be within the permissible limits of the standards (ASTM D-3751, EN14214) and petrodiesel comparative studies (Kumari et al., 2009; Garlapati et al., 2021). Moreover, FAME’s properties also depend on the fatty acid profiles of utilized oleaginous oils. The fatty acid profiles of oleaginous oils also resemble the edible and non-edible oils in prominent fatty acid presence such as C_16:0_, C_18:0_, C_18:1_, and C_18:2_ (Patel et al., 2017). The presence of unsaturated fatty acids is the ideal constituent for FAME’s production, up to certain tolerable polyunsaturated fatty acid presence limits. The unsaturation of produced FAMEs drawn through the determination of iodine value (IV) and polyunsaturated fatty acids dictate the oxidative stability of produced FAMEs. The formation of soaps through unreacted fatty acids in the produced FAMEs was dictated through the saponification value. The determination of kinematic viscosity will dictate the flow properties of FAMEs in the engine.

The high proportion of saturated fatty acids in FAMEs mitigates auto-oxidation, which eventually enhances the shelf life. In the meantime, unsaturated fatty acids in FAMEs are prone to cold-flow plugging properties (CFPP). Hence, it is recommendable to maintain an optimum ratio of saturated and unsaturated fatty acids in FAMEs (possible through the initial selection of single-cell oils) for better physicochemical properties (oxidative stability, better cold-flow properties) (Meng et al., 2009; Patel et al., 2017). Determination of IV facilitates the estimation of FAME’s unsaturated fatty acid content. The IV values of FAMEs need to be within prescribed limits to avoid gum formation through glyceride polymerization on heating (Knothe, 2006; Patel et al., 2016). The enhanced and smooth engine run is feasible with high cetane numbers, which denote better ignition and complete combustion of FAMEs with reduced gaseous and particulate emission profiles.

On the other hand, the high heating value (HHV) of FAMEs is usually on the higher end side, presumably with higher C/H and C/N ratios (Ramírez-Verduzco et al., 2012). The emissions and combustion problems of FAMEs also related to their high kinematic viscosity (KV), which results in large size of droplets and less combustion efficiency (Patel et al., 2017; Garlapati et al., 2021). The other property of FAMEs, namely, density, also has a detrimental effect on the air-to-fuel ratio and HHV. The KV and density of FAMEs need to be within permissible limits for fruitful engine performance (Hoekman et al., 2012).

The oxidative stability (OS) gives information about the auto-oxidation potential, which dictates FAME’s shelf life, which has to be preferential to be on the higher side. The FAME’s OS is usually linear with fatty acid chain length and has inverse behavior with double bonds in *cis* configuration (Hoekman et al., 2012; Stansell et al., 2012). Overall, the presence of unsaturation, soaps, particulate contaminants, and sulfur can influence the cetane number, HHV, and cold filter plugging point, which have to be determined to propose the suitability of the oleaginous FAMEs as transportation fuels (Patel et al., 2016). The fuel properties of oleaginous FAMEs determined by different researchers have been summarized in Table 6.

**TABLE 6:** Physico-chemical properties of lignocelullose-based oleaginous FAME’s.

Oleaginous microbes (Lignocellulosic substrate)	Fatty acid Profile (%)	Oxidative stability, at 110 ^*o*^C (h)	Kinematic viscosity (mm^2^/s at 40 ^*o*^C)	Cold filter plugging point (CFPP,^*o*^C)	Density (g/cm^3^ at 20 ^*o*^C)	Saponification value (mgKOH)	Iodine value (mg I_2_/100g)	Cetane number	High heating value (MJ/kg)	References
*Rhodotorula mucilaginosa* KKUSY14 (UDPH)	C_16:0_ –19.2 C_18:0_ –9.5 C_18:1_–51.2 C_18:2_—		4.85	7.62	0.873		50.53	66.52	41.41	Siwina and Leesing, 2021
*Rhodotorula mucilaginosa* KKUSY14 (UDPH)	C_16:0_ –18.2 C_18:0_ –2.4 C_18:1_ –65.7 C_18:2_ —		4.79	–6.86	0.878		59.77	61.33	40.68	Siwina and Leesing, 2021
*Mucor indicus* (CS)	C_16:0_ –13.7 C_18:0_ –53.5 C_18:2_ –6.1 C_18:3_–4.0	14.27	4.11	23.51	–	–	70	57.7	–	Alavijeh et al., 2020
*Rhodotorula taiwanensis* AM2352 (CH)	C_16:0_ –24.4 C_18:0_ –2.9 C_18:1_ –46.8 C_18:2_ –6.35	–	4.82	4.6	–	–	81.39	58.79	39.61	Miao et al., 2020
*Cryptococcus curvatus* (WOPH)	C_16:0_ –3.95 C_18:0_ –7.07 C_18:1_ –50.8 C_18:2_ –4.25	–	–	–4.13	0.92	222.09	79.06	53.09	41.61	Nair et al., 2020
*Bacillus cereus* (MF908505) (PWP)	–	–	4.6	–	0.88	–	115	–	–	Kanakdande et al., 2020
*Rhodotorula pacifica* INDKK (UPSH)	C_16:0_ –28.8 C_18:0_ — C_18:1_ –52.5 C_18:2_ -12.4 C_18:3_ –1.37		3.9	–6.1	0.87		86.29	53.92	38.14	Kumar et al., 2020b
*Rhodotorula pacifica* INDKK (DPSH)	C_16:0_ –14.4 C_18:0_ –6.13 C_18:1_ –61.9 C_18:2_ –16.8 C_18:3_ –1.3		3.7	–5.97	0.84		73.58	56.72	39.42	Kumar et al., 2020b
*Meyerozyma guilliermondii* (Bag)	–	0	–	–9	0.65	167.35	15.61	75.4	29.17	Ananthi et al., 2019
*Meyerozyma guilliermondii* (RH)	–	47.4	–	–9.54	0.82	225.78	39.27	61.64	36.08	Ananthi et al., 2019
*Pichia kudriavzevii* (Bag)	–	0	–	–16.48	0.88	472.54	0	57.85	30.41	Ananthi et al., 2019
*Pichia kudriavzevii* (RH)	–	0	–	–6.3	0.87	223.37	0	70.73	39.03	Ananthi et al., 2019
*Pichia manshurica* (Bag)	–	0		47.24	0.87	262.42	9.34	65	37.76	Ananthi et al., 2019
*Pichia manshurica* (RH)	–	0	–	–16.48	0.79	356.38	0	61.62	30.16	Ananthi et al., 2019
*Pichia kudriavzevii* (Bag)	–	10.95	–	9.88	0.56	130.99	40.95	78.75	24.8	Ananthi et al., 2019
*Pichia kudriavzevii* (RH)	–	37.17	–	–15.63	0.84	250.34	19.42	63.73	36.39	Ananthi et al., 2019
*Candida albicans* (Bag)	–	0	–	–16.48	0.87	247.64	0	68.34	38.26	Ananthi et al., 2019
*Candida albicans* (RH)	–	0	–	–16.48	0.87	331.93	0	62.74	35.17	Ananthi et al., 2019
*Rhodotorula mucilaginosa* (Bag)	–	22.34	–	51.1	0.74	183.38	24.46	70.59	33.27	Ananthi et al., 2019
*Rhodotorula mucilaginosa* (RH)	–	21.31	–	0.02	0.88	331.62	12.73	59.89	35.53	Ananthi et al., 2019
*R. mucilaginosa* Y-MG1 (WBH)	C_16:_ –11.9 C_18:0_ –11.0 C_18:1_ –66.1 C_18:2_ –7.1 C_18:3_ –0.8	–	1.39	–	0.86	–	75.45	56.68	–	Ayadi et al., 2019
*Wickerhamomyces anomalus* (AIW)	C_16:0_ –21.0 C_18:0_ –9.0 C_18:1_ –52.0 C_18:2_ –22.0	–	–	–0.54 to 10.4	–	190.69–203.13	61.77–88.32	53.45–59.32	–	Arous et al., 2017
*Cryptococcus psychro-tolerans* IITRFD (GSH)	C_16:0_ –29.4 C_18:0_ — C_18:1_ –37.8 C_18:2_ –32.8	6.2	3.75	–7.24	0.88	204.49	93.37	51.96	32.77	Deeba et al., 2017
*Rhodotorula mucilaginosa* IIPL32 (SB)	C_16:0_ –18.5 C_18:0_ –0.9 C_18:1_–38.1 C_18:2_–17.9	–	4.07		0.877	–	–	55.94	–	Khot and Ghosh, 2017
*R. mucilaginosa* IIPL32 (SBH)	C_16:0_–13.1 C_18:0_ – C_18:1_–55.5 C_18:2_ –18.9		3.79	–12.35	0.88		97.04	51.10	39.62	Dasgupta et al., 2017
Tropical Mangrove Fungus (SB)	C_16:0_ –0.4 C_18:0_ –0.5 C_18:1_ – 14.4 C_18:2_–36.8	-	4.12		0.87	202.38	104.2	52.1	39.57	Kakkad et al., 2015
*Rhodotorula glutinis* (LBH)	C_16:0_ –13.3 C_18:0_ –5.1 C_18:1_ –55.5 C_18:2_ –20.2	7.04	3.96	–3.21	0.88	197.12	99.86	48.52	39.85	Yen and Chang, 2015
*Rhodosporidium toruloides* 21167, (Cst)	C_16:0_ –21.6 C_18:0_ –5.8 C_18:1_ –51.6 C_18:2_ –17.7	9.25	4.24	0.81	0.87	191.43	74.86	55.72	40.46	Gen et al., 2014
*Rhodosporidium toruloides* 2F5 (Iln)	C_16:0_ –22.1 C_18:0_ –13.7 C_18:1_ –52.1 C_18:2_ –10.9	13.5	4.25	14.64	0.87	191.47	63.72	58.55	40.62	Wang et al., 2014
*Trichosporon cutaneum* (CAH)	C_16:0_ –12.5 C_18:0_ –6.6 C_18:1_ –72.1 C_18:2_ –5.6	23.64	4.53	–0.92	0.87	18.4	71.5	57.4	40.7	Chen et al., 2013
*Rhodotorula graminis* (CSH)	C_16:0_ –20.5 C_18:0_ –7.1 C_18:1_ –42.1 C_18:2_ –17.1	8.47	4.19	2.76	0.87	173.8	73.3	58.9	41.2	Galafassi et al., 2012
*Trichosporon dermatis* (HCC)	C_16:0_ –27.1 C_18:0_ –14.3 C_18:1_ –40.9 C_18:2_ – 9.9	14.50	4.52	17.21	0.87	179.3	52.0	63.4	41.2	Huang et al., 2012
*Y. lipolytica* (NDLH)	C_16:0_ –6 C_18:0_ –2 C_18:1_ –56 C_18:2_ –19.9	8.51	4.1	–10.5	0.86	158.0	72.0	58.0	41.5	Yu et al., 2011
*Y. lipolytica* (DLH)	C_16:0_ –5.7 C_18:0_ –0.8 C_18:1_ –55.3 C_18:2_ –20.9	8.23	4.1	–11.0	0.86	156.0	73.0	58.0	41.6	Yu et al., 2011
*C. curvatus* (NDLH)	C_16:0_ –25.9 C_18:0_ –15.2 C_18:1_ –47.7 C_18:2_ –6.4	20.95	4.2	18	0.86	185	46	60	40.9	Yu et al., 2011
*C. curvatus* (DLH)	C_16:0_ –27 C_18:0_ –15.3 C_18:1_ –45.0 C_18:2_ –7.3	18.74	4.25	18	0.86	183	47	60	41.0	Yu et al., 2011
*R. glutinis* (NDLH)	C_16:0_ –23.5 C_18:0_ –9.0 C_18:1_ –43.4 C_18:2_ –15.4	10.24	4.25	5	0.861	177	60	59	41.0	Yu et al., 2011
*R. glutinis* (DLH)	C_16:0_ –22.4 C_18:0_ –9.3 C_18:1_ –42.7 C_18:2_ –17.0	9.52	4.25	5	0.861	176	61	59	41.2	Yu et al., 2011
*Y. lipolytica* (DLH)	C_16:0_ –5.7 C_18:0_ –0.8 C_18:1_ –55.3 C_18:2_ –20.9	8.52	4.52	–13.16	0.87	153.17	82.43	60	41.95	Yu et al., 2011
*L. starkeyi* (DLH)	C_16:0_ –37.1 C_18:0_ –5.5 C_18:1_ –45.1 C_18:2_ –4.9	26.65	4.53	5.59	0.87	181.94	47.16	64.27	41.26	Yu et al., 2011

*DLH, detoxified liquid wheat straw hydrolysate; NDLH, non-detoxified liquid wheat straw hydrolysate; LBH, lignocellulosic biomass hydrolysate; SB, sugarcane baggase; HCC, hydrolysates of corncobs; CSH, corn stover hydrolysate; CAH, corncob acid hydrolysate; SBH, sugarcane bagasse hydrolysate; UDPH, undetoxified durian peel hydrolysate; CS, corn stover; CH, corncob hydrolysate; WOPH, waste office paper hydrolysate; PWP, pomegranate waste-peel; UPSH, undetoxified pongamia shell hydrolysate; DPSH, detoxified pongamia shell hydrolysate; Bag, baggase; RH, rice husk; WBH, wheat bran hydrolysate; AIW, agro-Industrial wastewaters; GSH, groundnut shell hydrolysate; CST, cassava starch; Iln, inulin.*

## Metabolic Engineering Approaches for Enhanced Lipid Synthesis and Biorefinery Products

Oleaginous microorganisms are gaining great interest for their ability in the production of target compounds using varied carbon sources (Kumar et al., 2020e). However, to exploit these OMs with high lipid productivity and yield at industrial scale, the strains need to be optimized. Synthetic biology, ME, genome editing, and genetic elements are being advanced; combining these essential components, OMs could be modified for biofuel production, industrial chemicals, and biorefinery. Strategies pertinent to ME of OMs are composed of (1) production of fatty acid-derived compounds [FAMEs, TAGs, fatty alcohols (FALs), FFAs]; (2) acetyl CoA-derived products [terpenoids, poly-3-hydroxybutyrate (PHB)]; (3) production of industrial compounds like citric acid, succinic acid, α-ketoglutarate, itaconic acid, and erythritol; (4) utilization of low-cost substrates such as LCB, starch, inulin, molasses, and glycerol (Levering et al., 2015).

All strategies are aimed for the development of viable technologies for either biofuel production or biorefinery compounds (Shi and Zhao, 2017). The last strategy has targeted to utilize the feedstocks efficiently in the fermentation process. As many strains are incapable of growing in inexpensive carbon sources, OMs need to engineer for efficient utilization of inexpensive carbon sources. Some efforts are being laid to utilize cellobiose, xylose from hemicelluloses, starch, molasses, inulin, and glycerol, which is a by-product of biodiesel industry. Similarly, model systems with targeted industrial chemicals would lead to biorefinery development. Although ME strategies are showing promising results, ME of oleaginous yeasts and microalgae is in its infancy. A plausible reason is low flux toward synthesis of target compounds due to low activity of heterologous pathways. Hence, future studies should target engineering of novel enzymes with stability and higher activity, specificity, optimization of heterologous and homologous pathways, and maintenance of balance between growth and lipid productivity (Tai and Stephanopoulos, 2013; Blazeck et al., 2014).

Synthetic biology approaches are promising and facilitate biological engineering of strain cycle through Design-Build-Test-Lean (DBTL) approach. Recent advances in synthetic biology research have facilitated developing new tools to perform genetic engineering in nonconventional yeasts such as *Yarrowia lipolytica* and *R. toruloides* (Bredeweg et al., 2017; Park et al., 2018; Xiong et al., 2021). Cloning of essential genetic elements such as constitutive, inducible, targeted, and repressible promoters is being carried out in *R. toruloides* and *Y. lipolytica* (Nora et al., 2019). Besides, engineering of tandem copies pertinent to upstream activation sequences (UASs) has been modified to strengthen the hybrid promoter for higher expressions in *Y. lipolytica* (Blazeck et al., 2011; Xiong and Chen, 2020). Genome editing mediated by clustered regularly interspersed short palindromic repeats (CRISPR) along with Cas protein has been studied in *Y. lipolytica* and *R. toruloides*, which can be optimized to improve the efficiency of lipid accumulation by adopting multiplex genome engineering with low off-target effects (Jiao et al., 2019; Otoupal et al., 2019; Schultz et al., 2019; Abdel-Mawgoud and Stephanopoulos, 2020; Yang et al., 2020). In addition to these approaches, Koh et al. (2014) developed Cre-*loxp* recombination system for marker-free transformation in *R. toruloides* and *Y. lipolytica* that could enhance the homologous frequency with the disruption of *Ku*70 encoding gene. Besides, to improve the stability and yield of oleaginous engineered strains, bacterial transcriptional factors coupled with genetically encoded biosensors pertinent to malonyl-CoA and flavonoid pathway have been recruited in *Y. lipolytica* (Lv et al., 2020). These studies imply that the tools for enhanced genetic engineering for increased lipid accumulation coupled with biorefinery in OMs could be a promising approach for the development of commercially viable technologies.

## Future Prospects for Integration of Biofuel Production From Lignocellulosic Biomass

The development of a viable process is an essential feature of a mature technology that can be done by adopting a circular economy approach. Unlike linear economy, circular economy generates value in at least four domains: (1) raw substance reutilization for biofuels with increased value; (2) enhances mutual economic growth through the development of liquid markets, where the products are exchanged among users; (3) integration of bioprocesses with zero waste creation; and (4) reusable potential of products that are applicable for varied purposes (Nogué et al., 2018; Chandel et al., 2020). In the bioethanol industry, conversion of LCB to bioethanol production has been done, but in terms of economic viability, several developed technologies are still in the infancy stage (Chintagunta et al., 2016; Jacob et al., 2016). Economic viability of the process could be ascertained through circular economy, where the by-product or waste can be reused that ultimately reduces the cost involved in the process (Amore et al., 2016; Chandel et al., 2020). Here, we are proposing a scheme of circular economy for biofuel production from LCB using OMs.

**FIGURE 2:**
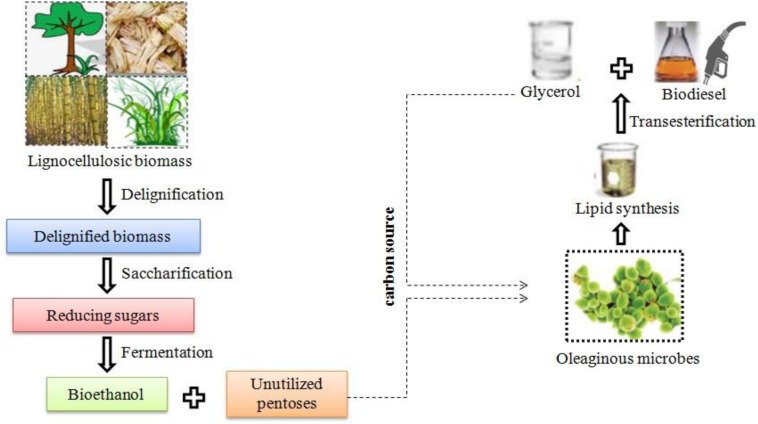
Schematic diagram of integrated biofuel production from lignocellulosic biomass using oleaginous microorganisms.

In bioethanol production, delignification, saccharification, and fermentation are the major steps involved in the process. After the delignification process, the lignin degraded particularly using enzymatic means can be used as antioxidants, glucose biosensor, reusable adsorbent, silver nanoparticles, electric double-layer capacitor, etc. (Kumar et al., 2020e). In the fermentation process, pentoses remain mostly unutilized. Hence, supplementing these pentoses as carbon source to OMs for lipid production could be a viable option. Furthermore, these lipids can be converted to FAMEs, i.e., biodiesel and glycerol, where the latter can be used as a carbon source for biodiesel production (Figure 2). The biomass generated after lipid extraction can be used for nutrient enrichment along with cyanobacteria and can be used as biomanure and biofertilizer, respectively. Therefore, integration of processes could lead to synthesis of biofuels and industrial chemicals with zero-waste concept.

## Conclusion

Biofuels play an important role in enabling society toward sustainable development. Development of economic biofuels particularly biodiesel can be achieved using LCB and OM owing to their capacity of utilizing inexpensive feedstocks for higher lipid accumulation. Furthermore, integration of biofuel process such as bioethanol and biodiesel production has great scope for the development of economic viable technologies that ultimately edify society. Economic viability of the process could be ascertained through life cycle assessment and techno-economic analysis studies.

## Author Contributions

AC reviewed the literature, drafted the manuscript, and drew the figures. GZ reviewed the literature and drafted the manuscript. MK reviewed the literature and compiled and drafted the manuscript. SK conceived the idea and analyzed and drafted the manuscript. VG drafted the manuscript and compiled the information. PP compiled the information and drafted the manuscript. NK edited the manuscript. AC meticulously analyzed, compiled, and edited the manuscript. JS-G compiled the literature and edited the manuscript.

## Conflict of Interest

The authors declare that the research was conducted in the absence of any commercial or financial relationships that could be construed as a potential conflict of interest.

## Publisher’s Note

All claims expressed in this article are solely those of the authors and do not necessarily represent those of their affiliated organizations, or those of the publisher, the editors and the reviewers. Any product that may be evaluated in this article, or claim that may be made by its manufacturer, is not guaranteed or endorsed by the publisher.
